# Selected single-nucleotide variants in *GRIN1*, *GRIN2A*, and *GRIN2B* encoding subunits of the NMDA receptor are not biomarkers of schizophrenia resistant to clozapine: exploratory study

**DOI:** 10.1007/s43440-020-00165-4

**Published:** 2020-10-06

**Authors:** Marek Krzystanek, Marek Asman, Joanna Witecka, Artur Pałasz, Ryszard Wiaderkiewicz

**Affiliations:** 1 Department and Clinic of Psychiatric Rehabilitation, Department of Psychiatry and Psychotherapy, Faculty of Medical Sciences, Medical University of Silesia in Katowice, Ziołowa 45/47, 40-635 Katowice, Poland; 2 Department of Parasitology, Faculty of Pharmaceutical Sciences in Sosnowiec, Medical University of Silesia in Katowice, Jedności 8, 41-200 Sosnowiec, Poland; 3 Department of Histology, Faculty of Medical Sciences, Medical University of Silesia in Katowice, Medyków 18, 40-752 Katowice, Poland

**Keywords:** Clozapine-resistant schizophrenia, NMDA receptor, Cognitive deficits, Single-nucleotide variants

## Abstract

**Background:**

Schizophrenia is a common mental illness whose pathogenesis is still unknown. The vulnerability and stress model in schizophrenia assume that susceptibility to the disease is mainly associated with genes. Of the five symptomatic dimensions of schizophrenia, cognitive impairment appears to be most associated with the pathogenesis of schizophrenia. The aim of the study was to explore whether selected nucleotide variants in *GRIN1*, *GRIN2A*, and *GRIN2B* encoding subunits of the *N*-methyl-d-aspartate receptor (NMDA-R) receptor occur in a selected group of patients with treatment resistant schizophrenia with cognitive impairment.

**Methods:**

The study included 45 patients diagnosed with super refractory schizophrenia, all with cognitive deficits and chronically psychotic. DNA fragments including the studied polymorphisms of the NMDA receptors subunit genes were amplified by polymerase chain reaction and subjected to sequencing.

**Results:**

The study did not confirm the presence of any of the four selected single-nucleotide variants in *GRIN1*, *GRIN2A*, and *GRIN2B* subunits of NMDA-R in the study group.

**Conclusion:**

Results of the study indicated that the selected single-nucleotide variants are not associated both with resistance to clozapine and the presence of cognitive deficits in

schizophrenia. It is possible, however, that a more extensive sequencing along with analyzing the expression of these genes may reveal different single-nucleotide variants than those assumed in the study.

## Introduction

Due to the unknown pathogenesis of schizophrenia, its treatment still faces great difficulties. Although resistance to treatment in schizophrenia is defined as the persistence of symptoms despite at least two antipsychotic treatments, with the assumption that drugs are taken by patients at an adequate dose and for an adequate time, it can occur from the first episode of schizophrenia [1]. Another problem is that although clozapine is an antipsychotic registered for a treatment of refractory schizophrenia, it is estimated that 60% of patients with treatment refractory schizophrenia do not even respond to clozapine. In the literature, this form of schizophrenia is referred to as super refractory schizophrenia (SRS) [2].

Difficulties in understanding the true causes of schizophrenia should be sought in the complexity of its pathogenesis. Great hopes for solving the puzzles of schizophrenia were associated with resequencing the human genome. Today, however, we know that the cumulative effect of single-nucleotide variants (SNVs) explains only about 30% of the genetic risk of developing schizophrenia [3].

The cause of schizophrenia may be indirectly related to its first symptoms, which would appear clinically and would reflect the onset of the pathogenetic process in the central nervous system. Because cognitive impairment occurs 3–4 years before the onset of schizophrenia, it could be associated with the pathogenesis of schizophrenia [4–6].

One of the elements of the putative pathogenesis of schizophrenia is disorder in the glutamatergic system [7, 8]. Inhibition of these receptors or more their dysfunction may be the cause of cognitive disorders in schizophrenia [9]. The insufficiency of N-methyl-d-aspartate receptor (NMDA-R) is closely associated with the appearance of cognitive dysfunctions and hypofrontality in schizophrenia [7]. However, this problem is more complex—administration of antipsychotic drugs alone may cause inhibition of NMDA glutamatergic receptor activity and cause secondary hypofrontality [10].

The results of pharmacogenetic studies may be of significant clinical importance in the treatment of schizophrenia. Genetic studies aim to identify diagnostic biological markers for schizophrenia, and pharmacogenetic studies to establish markers of efficacy and tolerance for antipsychotic drugs. In studies on clozapine, it was possible to identify single-nucleotide variants (SNVs) in the human leukocyte antigen (HLA)—the 6672G/C nucleotide variant, which is being tried to be used as a marker of the effectiveness of schizophrenia. The test had a high specificity; however, it was obsolete for commercial use due to low market interest [11]. Similarly, on daily basis practice, the testing of polymorphisms in CYP2D6 with regard to the metabolism of clozapine and determining its effective dose is used [12]. Since NMDA receptor dysfunction is a key component of the contemporary model of schizophrenia, identification of the nucleotide variants in NMDA receptor would reveal new markers of clozapine resistance in patients with schizophrenia. The discovery of such markers would allow faster decisions regarding the choice of treatment in schizophrenia.

The aim of the study was to look for genetic biomarkers of schizophrenia refractory to clozapine treatment in a group of patients corresponding to such a hypothetical clinical endophenotype. Single-nucleotide variants in *GRIN1*, *GRIN2A*, and *GRIN2B* encoding subunits of the NMDA receptor were selected as potential genetic biomarkers.

## Materials and methods

### Study group

**Fig. 1:**
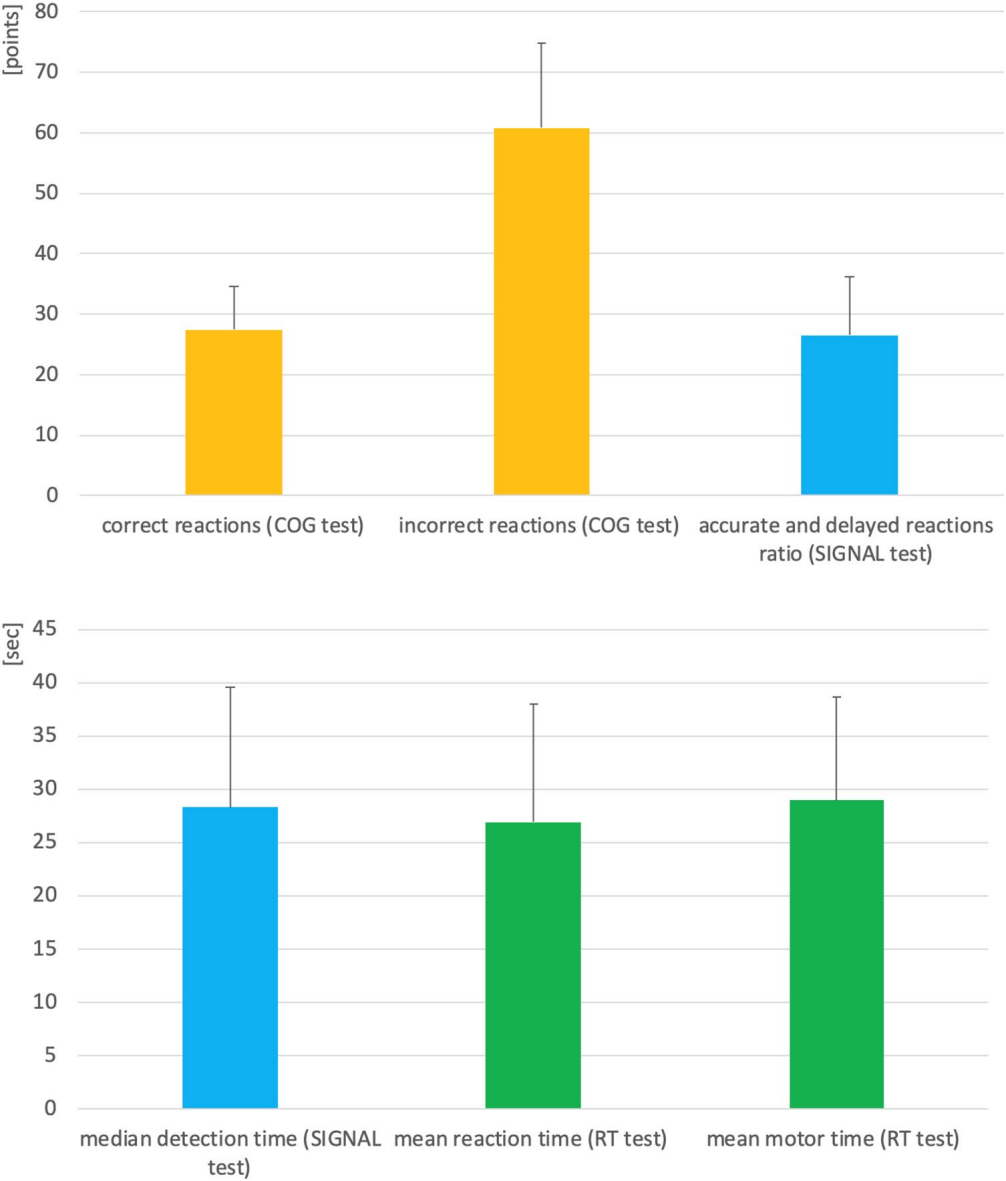
Cognitive performance in the study group measured with tests, belonging to the Vienna test system. Cognitron (COG) test assesses the concentration performance. Signal detection test (SIGNAL) measures long-term focused attention and the visual differentiation of a relevant signal when distractor signals are present. Reaction test (RT) is used to assess the ability to react under simple stimulus constellations (simple and choice reactions). The results are expressed on the ten scale (norm for the range of 40–60 points). Results in the bar graphs are expressed as mean + SD

45 patients diagnosed with schizophrenia (according to ICD-10 criteria and DSM-4) were selected for the study. The study was conducted on a targeted group of patients with super resistant schizophrenia. All the studied patient population had a common clinical endophenotype, for which the association was sought with the presence of SNVs in these patients. All patients were treated with antipsychotics at the time of the study, mostly except clozapine, because of the previously proven ineffectiveness of this drug. They were classified as schizophrenia resistant to clozapine treatment (SRS) as in the past and for at least 3 months each of them has been treated with clozapine at a therapeutic dose without improvement [13]. The presence of cognitive impairment was confirmed by neuropsychological examination using the Vienna test system (Fig. 1) [13]. Schizophrenia symptoms were assessed with PANSS scale. In all patients, psychotic symptoms were present (average total score was 89 points) and all of them showed significant cognitive impairment.

The characteristics of the study groups are summarized in Table 1.

**Table 1:** Description of the studied groups

Patients	Schizophrenia patients treatment super-resistance (*n* = 45)	Healthy volunteers (*n* = 30)
Gender		
F—female	F = 20	F = 16
M—males	M = 25	M = 14
Age (± SD^a^)	49.1 (± 9.9)	57 (± 7.3)
Min–max^b^ (years)	22–65	46–65
Age of onset (± SD)	27.4 (± 7.9)	NA^c^
Min–max^b^ (years)	18–45	
Duration of disease (± SD)	23.1 (± 11.5)	NA^c^
Min–max^b^ (years)	4–50	
Number of hospitalizations	11.7	NA^c^
Dose mg/day (average number of antipsychotics)	Different (1.6)	NA^c^

^a^Standard deviation

^b^Minimum and maximum

^c^Not applicable

### DNA isolation

The DNA was isolated from the peripheral blood of patients and healthy volunteers using the Blood Mini kit (A&A Biotechnology, Poland) according to the manufacturer's protocol. Concentrations of isolated samples were measured spectrophotometrically using Biomate 3 spectrophotometer (Thermo Scientiffic, USA) at wavelength 260/280 nm.

### DNA single-nucleotide variants detection and analyses

Single-nucleotide variants in NMDA-R subunits *GRIN1* (rs11146020) and *GRIN2B* (rs1806201) have been previously described in the literature and studied in various populations, as associated with schizophrenia or effectiveness of its treatment (Table 2) [14–17]. The MAF for the first one is only 0.1, but the OR is relatively high: 1.85 for heterozygotes (95% CI 1.43–2.42, *P* < 0.0001) and 2.46 for homozygotes (95% CI 1.17–6.84, *P* = 0.017) [18]. The variants in *GRIN2A* (NG_011812.2; Table 2) and *GRIN*2B (NG_031854.2; Table 2) were determined according to the NMDA-R protein structure available at the time when the research was conducted in The Universal Protein Resource database (https://www.ebi.uniprot.org) and proposed areas that may be of key importance for receptor function.

**Table 2:** Single-nucleotide variants (SNV’s) selected in the study with their position in reference sequence, minor allele frequency and allele frequency together with their putative function

Gene	dbSNP^a^ number/the location of the test site in the reference sequence/location in the gene	MAF^b^/AF^c^	Function
*GRIN1*	rs11146020/NG_011507.1:g.4476G>C/5′UTR	Global—0.107/0.08521 Europe—0.100/0.09817	Polymorphism associated with susceptibility to schizophrenia
*GRIN2A*	Lack of described SNP NM_00833.5: c.2304–2306 (AAT)/NG_011812.2:g.358165–358167 NP_000824.1:p.Asn614/Exon 10	Not applicable	Site determining NMDA receptor function
*GRIN2B*	rs1806201/NG_031854.2:g.422439C>T NP_000825.2:p.Thr888=/Exon 13	Global—0.303/0.2389 Europe 0.278/0.2679	Polymorphism associated with the effectiveness of schizophrenia treatment
*GRIN*2B	Lack of described SNP NM_000834.5: c.2055–2057 (AAC)/NG_031854.2:g378243-378245 NP_000825.2:p.Asn615/Exon 9	Not applicable	Site determining NMDA receptor function

^a^dbSNP—single-nucleotide polymorphism database of nucleotide sequence variation

^b^MAF—minor allele frequency

^c^AF—allele frequency, genome aggregate data base (gnomADv3)

The sequences including the studied polymorphisms of the NMDA receptors subunit genes were amplified by polymerase chain reaction (PCR) and analyzed using the ABI Prism 310 capillary sequencer (Applied Biosystem, USA) according to the Applied Biosystem protocol.

### Polymerase chain reaction (PCR)

Forward and reverse primers for PCR were designed based on the nucleotide sequences of the tested genes available in online databases (GeneBank) (Table 3).

**Table 3:** Primer sequences for studied genes of NMDA-R subunits

Gene	Forward primer	Reverse primer
*GRIN1* subunit NR1	ACGCGGTGACACGGACCCCTCTAACGT (56 °C)	TCTGTTCGTGTACATGCGTGTGAATGAC (57.8 °C)
*GRIN2A* subunit NR2A	GGGCAATCACAGGACACAACTATC (57.1 °C)	CAGATGGAGAGGAAAGCAAGGTGA (57.1 °C)
*GRIN2B* subunit NR2B	AGTTCAAGAAAGACCATCCTACA (54.4 °C)	AAAACATAAGAAAGAACGGTCAAT (54.4 °C)
*GRIN2B* subunit NR2B	TGCCCACTTCCAACTCCTACTTAC (56 °C)	CATGATGTGGTTTCTTGCTTGAG (56 °C)

()—the optimal annealing temperature is given in brackets

The PCR reaction was carried out in a 50 µl volume containing 200 ng of DNA, Fast Start Taq polymerase (Roche, Germany), 0.25 pmol of each of the primers (Sigma-Aldrich, Poland), a buffer containing MgCl_2_ and 10 mM PCR nucleotide Mix (dNTP’s) (Roche, Germany). The final sample volume was adjusted to 50 µl with deionized water. The amplification reaction was carried out in a Mastercycler thermocycler (Eppendorf, Germany). The samples were denatured initially for 5 min at 94 °C, followed by 29 cycles in the following steps:

- proper denaturation for 30 s at 94 °C;
- annealing for 1 min, at a temperature depending on the starter used (Table 3);
- elongation for 1.5 min. at 72 °C.

The final elongation time was 5 min at 72 °C. At the final stage, the samples were cooled to 4 °C.

The 5 µl of the PCR product was separated on 2% ethidium bromide stained agarose gels. The gels were then visualized in ultraviolet light and analyzed using Kodak 1D Image Analysis 3.6 Software.

The bulk (45 µl) of the PCR product was incubated with Shrimp Alcaline Phosphatase and Exonuclease I (Fermentas, Lithuania) at 37 °C for 15 min and then at 80 °C for 15 min. Sequential PCR was then performed separately for each of the primer pairs using the BigDye Terminator v3.1 Cycle Sequencing Kit (Applied Biosystems, Germany) according to the protocol. The mixture was incubated in a thermocycler for 25 cycles including: denaturation of 96 °C for 30 s, annealing of 50 °C for 15 s, elongation of 60 °C for 3 min. and cooled at 4 °C. At the end of the test, alcohol precipitation was performed, then the samples were dried and lyophilized using Speed-Vac (Labconco, USA), and the resulting product was dissolved in 20 µl of Hi-Di Formamide solution (Applied Biosystem, USA). The samples sequences were analyzed using ABI Prism 310 capillary analyzer (Applied Biosystem, USA), and obtained results were verified for aligment using MegAlign software, DNAStar 5.0 Software (Lasergene, USA).

### Ethical issues

The study protocol was approved by the Bioethics Committee of the Medical University of Silesia in Katowice No. NN-6501-129/05. Blood samples for genetic procedures have been collected since 2007 and systematically sequenced up to 2017. The long period of collecting research material was caused by the difficulty in gathering a group of chronically psychotic patients with SRS and the even greater problem of obtaining their consent to participate in the study.

### Statistical methods

Descriptive statistics were used in the description of the study groups. Means and standard deviation as well as range of results were used in the calculations. The G*Power program (version 3.1.9.2) was used to perform power analysis [19].

## Results

Sequencing of the DNA fragments from schizophrenic patients and healthy control group was conducted to search for a polymorphism in the gene subunit *GRIN1* (rs11146020) associated with susceptibility to schizophrenia, *GRIN2B* (rs1806201) associated with the efficacy of neuroleptic and polymorphisms in genes of subunits, which determine NMDA receptor functions—*GRIN2A* [2304–2306 (AAT)] and *GRIN2B*—2055–2057 (AAC).

In the studied fragments of human DNA sought SNVs were not detected, both in the control group of healthy individuals as well as in the examined group of patient’s refractory to clozapine treatment.

**Fig. 2:**
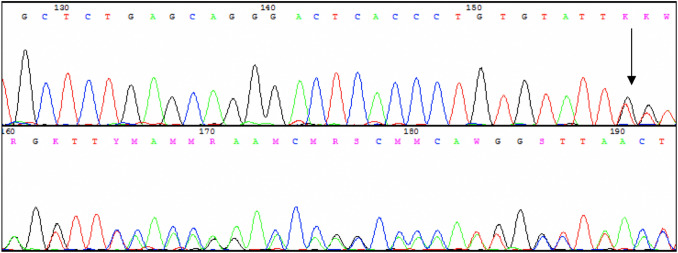
Example of sequencing a fragment of the *GRIN2B* subunit gene with a T deletion. The deletion site (rs56069446) is indicated by an arrow

At the same time, the sequences surrounding target SNVs were analyzed. SNV rs56069446 was found in the intron 8 of the *GRIN2B* gene, present in one healthy person in the control group and two people with non-remission schizophrenia (Fig. 2). This deletion has not been described as clinically relevant to date. The incidence of MAF in the general population is estimated at 0.024 [0.0572 according to genome aggregate database (gnomADv3)] and in the European population at 0.075 (0.08961 according to gnomADv3). This change is not yet described, and data on the occurrence of this change are derived from population sequencing (1000 genomes). Because of the low allele frequency, no statistical analysis was performed.

Power analysis was performed for two SNVs for which MAFs in the European population are known (Table 2). For the SNV in rs1806201 (*GRIN2B* gene), the MAF value for the European population is 0.278; therefore, we assumed the effect size 0.3 in the calculations. To achieve the test power of 0.8 at the significance level *α* = 0.05 and the assumption of a correlation factor of 0.3, the required size of the research group is 64 patients. For our group of 45 patients, under these assumptions, the power of the test is 0.66. In turn, for the SNV in rs11146020 (*GRIN1* gene) the MAF value is 0.1, so in the calculations we assumed the effect size 0.1. To achieve the test power of 0.8 with the significance level *α* = 0.05 and the assumption of the correlation factor 0.1, the required size of the research group is 614 patients. For the group of 45 patients, under these assumptions, the power of the test is 0.16.

## Discussion

The study conducted on the SNVs in NMDA receptor’s subunits was innovative and aimed at identifying new variants as markers of schizophrenia refractory to the treatment with clozapine. The possible existence of functional nucleotide variants in NMDA receptor subunits is indicated by the previously described cases of mutations in people with various neuropsychiatric symptoms. Nucleotide variants present in NMDA receptor subunits coexisted with language problems, hypotonia and ASD diagnosis [20]. The choice of SNVs in our study was determined by the previous association studies in schizophrenia and the analysis of the structure of the receptor itself. As previous studies on schizophrenia did not relate variants to the clinical endophenotype of ineffectiveness on treatment with clozapine, the conducted research was innovative.

Power analysis showed that the group of 45 people is very small to conduct the association study. If we were to conduct an associative study in a group of patients with schizophrenia, we would have to gather a sufficiently large study group related to the frequency of SNVs data in the population. We approached this issue from the other side. We assembled a targeted study group with a current clinical endophenotype, and in this population, we assumed the presence of SNVs in most of the subjects. Our analysis of these SNVs did not show such a relationship.

The presented study was based on the selection of a homogeneous group of patients without remission and with significant cognitive impairment, in whom treatment with clozapine was ineffective. The association of this clinical endophenotype with the single-nucleotide variants selected for study in the NMDA GRIN1, GRIN2A, and GRIN2B receptor coding subunits, would suggest that these SNVs could be potential markers of clozapine resistance in patients with schizophrenia. Our study did not confirm this possibility. However, we cannot exclude the existence of other SNVs present in the NMDA receptor subunits tested that may be associated with clozapine-resistant schizophrenia. It is possible that carrying out more extensive sequencing along with analyzing the expression of these genes would help answer this question.

It should be assumed that both the phenomenon of resistance to clozapine treatment and the occurrence in this group of cognitive impairment are associated with a different genetic background or the influence of environmental factors. The patients in the study group took various antipsychotic drugs; therefore, the observed cognitive impairment may also be the effect of the antipsychotic drugs themselves [21].

The size of the study group as for genetic testing is the major limitation of the study, so the obtained results should be treated as preliminary and should be confirmed in bigger sample studies; however, in our opinion, if super-resistance to the treatment or cognitive impairment would be associated with the tested single-nucleotide variants, they should be present at least in some patients.

Our conclusion is that selected single-nucleotide variants in *GRIN1*, *GRIN2A*, and *GRIN2B* genes of NMDA-R do not seem to be associated with both resistance to schizophrenia treatment with clozapine, and also with the occurrence of cognitive disturbances in schizophrenia.
